# Prehospital triage of patients diagnosed with perforated peptic ulcer or peptic ulcer bleeding: an observational study of patients calling 1-1-2

**DOI:** 10.1186/s13049-018-0494-1

**Published:** 2018-04-05

**Authors:** Kasper Bonnesen, Kristian D. Friesgaard, Morten T. Boetker, Lone Nikolajsen

**Affiliations:** 1 Research Department, Prehospital Emergency Medical Services, Olof Palmes Allé 34, 8200 Aarhus N, Central Denmark Region Denmark; 2 Department of Anaesthesiology and Intensive Care, Aarhus University Hospital, Aarhus, Denmark

**Keywords:** Perforated peptic ulcer, Peptic ulcer bleeding, Prehospital, Triage

## Abstract

**Background:**

Triage systems are used in emergency medical services to systematically prioritize prehospital resources according to individual patient conditions. Previous studies have shown cases of preventable deaths in emergency medical services even when triage systems are used, indicating a potential undertriage among some conditions. The aim of this study was to investigate the triage level among patients diagnosed with perforated peptic ulcer (PPU) or peptic ulcer bleeding (PUB).

**Methods:**

In a three-year period in Central Denmark Region, all patients hospitalized within 24 h after a 1-1-2 emergency call and who subsequently received either a PPU or a PUB (hereinafter combined and referred to as PPU/PUB) or a First Hour Quintet (FHQ: respiratory failure, stroke, trauma, cardiac chest pain, and cardiac arrest) diagnosis were investigated. A modified Poisson regression was used to estimate the relative risk of receiving the highest and lowest prehospital response level. Also, a linear regression analysis was used to estimate the relative risk of 30-day mortality.

**Results:**

Of 8658 evaluated patients, 263 were diagnosed with PPU/PUB. After adjusting for relevant confounding variables, patients diagnosed with PPU/PUB were less likely to receive ambulance transportation compared to patients diagnosed with stroke, RR = 1.41 (CI: 1.28–1.56); trauma, RR = 1.28 (CI: 1.15–1.42); cardiac chest pain, RR = 1.47 (CI: 1.33–1.62); and cardiac arrest, RR = 1.44 (CI: 1.31–1.42). Among patients diagnosed with PPU/PUB, 6.5% (CI: 3.3–9.7) did not receive ambulance transportation. The proportion of patients not receiving ambulance transportation was higher among patients diagnosed with PPU/PUB compared to patients diagnosed with an FHQ diagnosis. The 30-day mortality rate among patients diagnosed with PPU/PUB was 7.8% (CI: 4.2–11.1). This was lower than the 30-day mortality rate among patients diagnosed with respiratory failure (*P* = 0.010), stroke (*P* = 0.001), and cardiac arrest (*P* < 0.001), but comparable to the 30-day mortality among patients diagnosed with cardiac chest pain (*P* = 0.080) and trauma (*P* = 0.281).

**Conclusion:**

Among patients calling 1-1-2, fewer patients diagnosed with PPU/PUB received ambulance transportation than patients diagnosed with FHQ diagnoses, despite a high mortality among patients diagnosed with PPU/PUB.

## Background

Triage systems are used in emergency medical services (EMS) for systematic prioritization of prehospital resources according to the presumed severity and urgency of the individual patient’s condition. Rapid diagnostics and treatment play a key role in severe time-critical conditions. Thus, avoiding undertriage is important. The criteria-based Danish Index for emergency care is used after all emergency calls (1-1-2) to an emergency medical communication center (EMCC) in Denmark. The Danish Index generally triages patients with the highest hospital admission risks and case fatality rates to the highest level of emergency [1]. A previous Danish study suggested cases of preventable deaths in EMS despite applying the Danish Index, thus indicating a potential undertriage among some conditions [2]. This has also been demonstrated in a Finnish EMS system [3]. The First Hour Quintet (FHQ) diagnoses (respiratory failure, stroke, trauma, cardiac chest pain, and cardiac arrest), are highly targeted in prehospital triage systems [4]. However, identifying other high-risk diseases may discover conditions susceptible for undertriage.

Diseases of the digestive system account for about 3% of diagnoses among patients hospitalized after a 1-1-2 call. However, they rarely receive the highest-level response (i.e. ambulance with lights and sirens) [1, 5]. Patients with the severe condition perforated peptic ulcer (PPU) have a 30-day mortality rate of more than 20%, and for patients with peptic ulcer bleeding (PUB) the rate is nearly 10% [6, 7]. To reduce mortality, national inhospital guidelines implemented nationwide recommend reduced time from hospital admission to diagnosis and treatment for these patients [8, 9]. However, versatile symptomatology (e.g. referred abdominal pain, anemia-caused tiredness, etc.) might lead to inadequate symptom description [10], making it difficult to raise a correct suspicion of PPU and PUB (hereinafter combined and referred to as PPU/PUB) after the medical emergency call. A combination of high short-term mortality and low prehospital triage level of patients with PPU/PUB may suggest a need for improvements in emergency care for these patients.

Therefore, the aim of this study was to examine prehospital triage among patients diagnosed inhospitally with PPU/PUB and compare these findings with the triage among other severe time-critical conditions. We hypothesize that patients diagnosed with PPU/PUB are less likely to receive the most acute level of triage despite mortality rates comparable to FHQ patients.

## Methods

### Study population and setting

This was a population-based observational study performed in the Central Denmark Region in a three-year period from December 1, 2011 to November 30, 2014. The Central Denmark Region covers an area of 13,007 km^2^ urban, suburban, and rural land with 1.3 million inhabitants corresponding to 23% of the Danish population [11].

We included all first time 1-1-2 calls within the study period. Exclusion criteria included: invalid civil personal register (CPR) number, if patients called more than one time in the study period, and non-existing symptom categories within the dispatch protocol. The cohort was identified via technical dispatch software in the EMCC, containing data on 1-1-2 calls, triage level, and prehospital time stamps. Vital status, gender, and age were retrieved from the Danish Civil Registration System [12], and data on previous diseases and present diagnoses, according to the 10th version of the International Classification of Disease (ICD-10), were retrieved from the National Patient Register [13]. Each Danish citizen has a unique CPR number that makes it possible to link Danish registers on an individual level. Patients were followed from hospital admission date to either death, emigration, or November 30, 2014 – whichever came first. The study was approved by the Danish Data Protection Agency (record number 1–16–02-207-15). Approval by the local ethics committee and collection of informed consent are not required for observational studies.

### Triage

The Danish health care system provides free and unconstrained access for all citizens to general practitioners, prehospital emergency medical services, and hospitals [14]. Thus, patients diagnosed with PPU/PUB or a FHQ diagnosis can gain access to acute medical help either via general practitioners, through the Danish national emergency number 1-1-2, or by appearance at a hospital (which is rare). When people dial 1-1-2, they are connected to a healthcare professional in the EMCC. In the Central Denmark Region, the EMCC is staffed by registered nurses and paramedics with six weeks’ additional training in communication and use of the dispatch protocol Danish Index [15]. This tool is designed to evaluate the severity and urgency of the patients’ conditions. It is divided into 37 symptom chapters (e.g. non-traumatic bleeding, stomach or back pain, traffic accident, etc.), each one subdivided into 5 levels of decreasing emergency (A-E).

PPU/PUB was defined according to specific ICD-10 diagnosis-codes listed by the Danish Clinical Register of Emergency Surgery [16]. Respiratory failure, stroke, cardiac chest pain, and cardiac arrest were defined according to specific ICD-10 diagnosis-codes listed by the European Emergency Data Project [17]. Trauma was defined as ICD-10 trauma diagnoses with an inhospital survival probability ≤0.941 based on pooled data from nearly 4 million injuries in seven industrialized countries, including Denmark, with similar emergency care setups [18, 19]. No validation of the trauma diagnoses has been conducted whereas all other diagnoses have been validated previously [20, 21]. The exact definition of each group is listed in the Appendix 3 (Table 5).

### Statistical analyses

All statistical analyses were conducted in STATA version 14.1 (StataCorp, TX, USA). Categorical data were presented as number and percentage (%) with a 95% confidence interval (CI). Comparisons of categorical data were made by a chi-squared test. Continuous data were presented as means with a 95% CI for normally distributed data and as medians with interquartile ranges (IQR) for skewed data. Comparisons of continuous data were made using a student’s *t* test or a Mann-Whitney U test when appropriate.

A modified Poisson regression with a robust error-variance approach was used to estimate the relative risks (RR) of level A triage and level E triage [22]. PPU/PUB was used as a reference point and the following covariates were included in the adjusted analyses: age, sex, Charlson Comorbidity Index (CCI) score, and time of 1-1-2 call.

30-day mortality was used as proxy of disease severity. The initially intended use of a Cox proportional regression analysis was abandoned, as the data did not fulfill the proportional hazards assumption. Instead, a generalized linear regression of pseudo-observations was conducted to achieve relative risk estimates of mortality at specific time points. This kind of statistics does not require the fulfillment of the proportional hazards assumption and was therefore applicable [23]; the primary analysis was conducted on complete cases. In a sensitivity analysis, we repeated the regression analysis on triage level after imputing missing data by following two models: a multiple imputation model using chained equations and a bootstrapping model [24, 25].

## Results

**Fig. 1:**
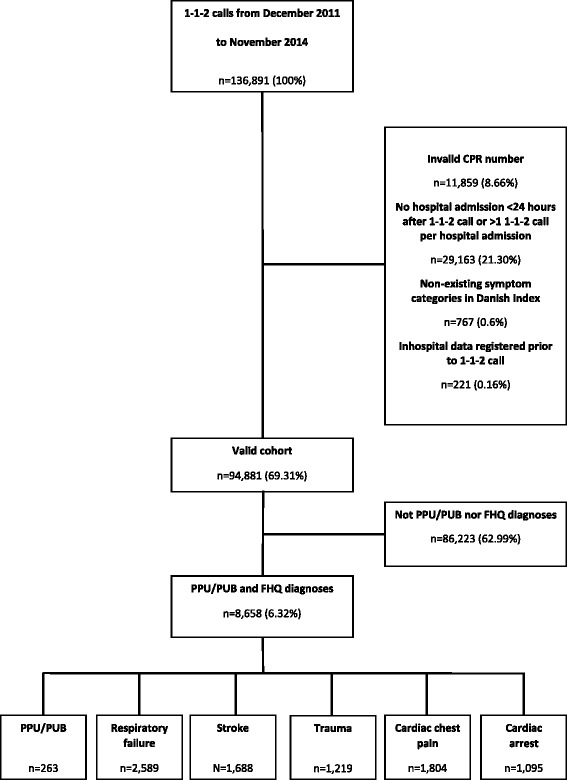
Flowchart of patient inclusion

In the three-year study period, 136,891 1-1-2 emergency calls were received by the EMCC in the Central Denmark Region. Figure 1 displays a flowchart of the patient inclusion. Of the 94,881 patients who fulfilled the inclusion criteria, 8658 (6.32%) were diagnosed with PPU/PUB or a FHQ diagnosis. Patients diagnosed with PPU/PUB were older and had more comorbidities than the average FHQ patient. The variation of potential confounding variables between PPU/PUB and FHQ diagnoses is displayed in Table 1. Among the 8658 patients investigated, 263 were diagnosed with PPU/PUB. Of these, 63.4% (CI: 57.1–69.6) received a level A response. After adjusting for age, sex, CCI score, and time of 1-1-2 call, the study showed that patients diagnosed with PPU/PUB were less likely to receive a level A triage compared to patients diagnosed with stroke, RR = 1.41 (CI: 1.28–1.56); trauma, RR = 1.28 (CI: 1.15–1.42); cardiac chest pain, RR = 1.47 (CI: 1.33–1.62); and cardiac arrest, RR = 1.44 (CI: 1.31–1.42). On the contrary, the risk of level E triage was higher among patients diagnosed with PPU/PUB compared to patients diagnosed with a FHQ diagnosis except for patients diagnosed with respiratory failure, (RR = 0.60 (CI: 0.35–1.05), Table 2). The association between diagnosis-groups and triage was not noticeably modified by age, sex, and CCI score (Table 3 in Appendix 1). After imputing missing data on triage level, patients diagnosed with PPU/PUB had a lower risk of receiving a level A triage compared to all FHQ diagnoses including respiratory failure. After imputation the results regarding level E triage remained robust, except in the bootstrapping model, where patients diagnosed with stroke had a comparable possibility of receiving a level E triage (see Table 4 in the Appendix 2).

**Table 1:** Baseline characteristics

Variable	PPU/PUB (*n* = 263)	Respiratory Failure (*n* = 2589)	Stroke (*n* = 1688)	Trauma (*n* = 1219)	Cardiac Chest Pain (*n* = 1804)	Cardiac Arrest (*n* = 1095)	Total (*n* = 8658)
Age, years (95% CI)	69.4 (67.6;71.2)	63.2 (62.3;64.1)	70.1 (69.4;70.8)	39.9 (38.7;41.1)	69.2 (68.5;69.8)	68.9 (68.0;69.0)	63.4 (63.0;63.9)
Sex, % (95% CI)							
Male	58.9 (53.0;64.9)	55.2 (53.4;57.2)	55.6 (53.3;58.0)	61.6 (58.8;64.3)	66.6 (64.4;68.8)	65.6 (62.8;68.4)	60.0 (59.0;61.0)
Emergency calls^a^, % (95% CI)							
1	95.1 (92.4;97.7)	89.5 (88.3;90.6)	95.6 (94.6;96.6)	98.5 (97.8;99.2)	89.9 (88.5;91.3)	98.1 (97.3;98.9)	93.3 (92.8;93.8)
2	4.9 (2.3;7.6)	7.4 (6.4;8.4)	4.0 (3.1;5.0)	1.3 (0.7;2.0)	8.3 (7.0;9.5)	1.7 (1.0;2.5)	5.3 (4.8;5.7)
3	–	1.7 (1.2;2.2)	0.3 (0.0;0.6)	0.1 (0.0;0.2)	1.2 (0.7;1.7)	0.2 (0.0;0.4)	0.8 (0.6;1.0)
4+	–	1.5 (1.0;1.9)	0.1 (0.0;0.2)	0.1 (0.0;0.2)	0.7 (0.3;1.1)	–	0.6 (0.4;0.8)
CCI score, % (95% CI)							
0	0.8 (0.0;1.8)	15.7 (14.3;17.1)	–	77.1 (74.8;79.5)	9.2 (7.9;10.5)	31.5 (28.8;34.3)	21.5 (20.6;22.3)
1	41.1 (35.1;47.0)	25.7 (24.0;27.4)	51.1 (48.8;53.5)	10.3 (8.5;12.0)	29.4 (27.3;31.5)	20.9 (18.5;23.3)	29.1 (28.2;30.1)
2	16.7 (11.2;21.3)	15.2 (13.8;16.6)	14.6 (12.9;16.3)	5.2 (3.9;6.4)	18.5 (16.7;20.3)	15.4 (13.3;17.6)	14.4 (13.7;15.2)
3+	41.4 (35.5;47.4)	43.4 (41.5;45.3)	34.2 (32.0;36.5)	7.4 (5.9;8.9)	43.0 (40.7;45.2)	32.1 (29.4;34.9)	35.0 (34.0;36.0)
Time of 1–1-2 call, % (95% CI)							
00:00–05:59	18.3 (13.6;22.9)	21.7 (20.1;23.3)	6.9 (5.7;8.1)	9.7 (8.0;11.3)	18.0 (16.2;19.7)	12.3 (10.4;14.3)	15.0 (14.3;15.8)
06:00–11:59	30.4 (24.8;36.0)	32.1 (30.3;33.9)	36.0 (33.7;38.3)	27.2 (24.7;29.7)	29.8 (27.7;31.9)	37.5 (34.7;40.4)	32.3 (31.4;33.3)
12:00–17.59	31.9 (26.3;37.6)	22.2 (20.6;23.8)	35.2 (32.9;37.5)	40.0 (37.3;42.8)	28.3 (26.2;30.4)	28.8 (26.1;31.5)	29.6 (28.7;30.6)
18:00–23:59	19.4 (14.6;24.2)	24.0 (22.4;25.7)	21.9 (19.9;23.9)	23.0 (20.6;25.3)	24.0 (22.0;26.0)	21.4 (18.9;23.8)	23.0 (22.1;23.9)

*Abbreviations*: *PPU* perforated peptic ulcer, *PUB* peptic ulcer bleeding, *n* number of patients, *CI* confidence interval, *CCI* Charlson Comorbidity Index

^a^Number of emergency calls per patient

**Table 2:** Triage and mortality

	Group	Frequency, % (95% CI)	Unadjusted, RR (95% CI)	Adjusted^a,b^, RR (95% CI)	*p*-value
A-triage (*n* = 7538)	PPU/PUB	63.26 (57.12–69.61)	1 (ref.)	1 (ref.)	–
	Respiratory Failure	69.73 (67.85–71.62)	1.10 (0.99–1.22)	1.09 (0.98–1.20)	0.105
	Stroke	89.22 (87.67–90.78)	1.41 (1.27–1.56)	1.41 (1.28–1.56)	< 0.001
	Trauma	83.81 (81.46–86.16)	1.32 (1.19–1.46)	1.28 (1.15–1.42)	< 0.001
	Cardiac Chest Pain	93.22 (91.99–94.45)	1.47 (1.33–1.62)	1.46 (1.33–1.62)	< 0.001
	Cardiac Arrest	92.23 (90.52–93.95)	1.46 (1.32–1.61)	1.44 (1.30–1.59)	< 0.001
E-triage (n = 7538)	PPU/PUB	6.47 (3.28–9.65)	1 (ref.)	1 (ref.)	–
	Respiratory Failure	3.79 (3.01–4.58)	0.59 (0.35–1.00)	0.60 (0.35–1.05)	0.073
	Stroke	2.56 (1.77–3.36)	0.40 (0.22–0.71)	0.41 (0.23–0.73)	0.002
	Trauma	0.74 (0.19–1.29)	0.11 (0.05–0.28)	0.13 (0.05–0.36)	< 0.001
	Cardiac Chest pain	1.62 (1.00–2.24)	0.25 (0.13–0.47)	0.25 (0.13–0.47)	< 0.001
	Cardiac Arrest	1.17 (0.50–1.86)	0.18 (0.08–0.39)	0.18 (0.08–0.40)	< 0.001
30-day mortality (n = 7538)	PPU/PUB	7.83 (4.52–11.13)	1 (ref.)	1 (ref.)	–
	Respiratory Failure	12.16 (10.87–13.44)	1.55 (1.01–2.39)	1.67 (1.06–2.64)	0.028
	Stroke	16.83 (15.01–18.66)	2.15 (1.39–3.32)	2.03 (1.27–3.24)	0.003
	Trauma	3.16 (2.16–4.17)	0.40 (0.24–0.68)	1.33 (0.72–2.44)	0.364
	Cardiac Chest Pain	4.45 (3.49–5.42)	0.57 (0.35–0.91)	0.64(0.38–1.06)	0.080
	Cardiac Arrest	47.34 (40.67–54.00)	6.05 (3.89–9.41)	6.72 (4.21–10.73)	< 0.001

*Abbreviations*: *PPU* perforated peptic ulcer, *PUB* peptic ulcer bleeding, *CI* confidence interval, *RR* relative risk, *n* number, *ref*. reference, *CCI* Charlson Comorbidity Index

^a^Triage adjusted for age, sex, CCI score, and time of 1–1-2 call

^b^30-day mortality adjusted for age, sex, CCI score, and time of 1–1-2 call – an interaction term between exposure and sex was included

The 30-day mortality rate among patients diagnosed with PPU/PUB was 7.8% (CI: 4.2–11.1). In the adjusted analysis, 30-day mortality was similar for patients with cardiac chest pain, RR = 0.64 (CI: 0.38–1.06) and patients diagnosed with trauma, RR = 1.33 (CI: 0.72–2.44), but higher for respiratory failure, RR = 1.67 (CI: 1.06–2.64); stroke, RR = 2.03 (CI: 1.27–3.24), and cardiac arrest, RR = 6.72 (CI: 4.21–10.73).

## Discussion

In this large observational study, including 8658 patients hospitalized within 24 h from a 1-1-2 call and diagnosed with PPU/PUB or a FHQ diagnosis, we found that the 30-day mortality rate among PPU/PUB patients was comparable to two of the five FHQ groups but fewer patients with PPU/PUB received level A triage compared to the FHQ patients.

The main objective of the EMCC is to dispatch the correct level of triage to keep the degree of undertriage to a minimum, as undertriage is associated with increased mortality. Andersen et al. discovered 18 potentially preventable deaths same day as the 1-1-2 call in an 18-month period [2]. Kuisma et al. also discovered 29 potentially avoidable deaths and one definitely avoidable death in patients receiving the lower urgency triage categories in a three-year period [3] and other studies have shown similar results [26–29]. The possibility of reducing the number of potential cases of undertriage seems present according to previous studies. A Belgium study has shown that two training sessions can increase the sensitivity of sending a mobile critical care unit along with a basic life support ambulance from 36% to 60% without any change in specificity [30]. Another Belgium study suggested trends towards an increased ability to obtain information from emergency callers among the telephone responders after a training session [31]. An American study showed that appropriate performance feedback could increase dispatch protocol compliance from 76% to 95% [32].

Today, the telephone responders in the EMCC are offered continuous education on a regular basis. However, a more systematic approach might be beneficial. The high amount of level A triage among patients diagnosed with cardiac chest pain (primarily acute myocardial infarction and angina pectoris) seen in our study might be an effect of previous research regarding patients diagnosed with ST-elevation myocardial infarction, causing a change in procedure for these patients. Today, ST-elevation myocardial infarction is diagnosed prehospitally and the patients are field-triaged directly to an invasive center. This has resulted in reduced time consumption of reperfusion, a decrease in mortality, and a lower risk of congestive heart failure [33–36]. On the contrary, increased attention to patients presenting themselves with chest pain may potentially result in overtriage [37, 38].

The main strength of this study is its large-sized population-based cohort, which improves precision and external validity. The unique CPR numbers provide the possibility of record linkage of validated registers on an individual level [12, 39]. Another strength is the free access to health care for all patients mitigating the risk of selection bias. Other studies might have an underrepresentation of less critical illnesses due to treatment expenses. Furthermore, diagnoses are validated for most ICD-10 diagnoses and this ensures correct classification. The nearly complete data set of the included covariates is also a strength. On the other hand, the weaknesses of this study relate to its register-based observational design. First of all, the proportion of patients with invalid CPR contributes to potential selection bias. However, missing data on emergency patients making a 1-1-2 call is difficult to avoid, as seen in other similar observational prehospital studies [1, 4, 40–42]. Compared to the final cohort, patients with invalid CPR had a higher proportion of level E triage and tended to call more frequently in the evening and at night. Further information on these patients was unobtainable, thus it remains unanswered whether these patients differed from the final cohort in other ways. Second, missing outcome data on triage level, especially among patients diagnosed with trauma, may eventually lead to bias. In order to address this issue, a sensitivity analysis with imputed triage level was conducted using two different imputation models, and no major estimate changes were observed. Last, although adjusted for several potential confounders, unobserved confounding would still be able to affect our estimates, and residual confounding cannot be ruled out.

Based on the current study, undertriage seems present among PPU/PUB patients, possibly caused by the versatile symptomatology, the caller’s description of the symptomatology, the telephone responders’ perception of the symptomatology, and the structure of the Danish Index. No prehospital guidelines exist explaining how to handle these patients. In the hospital setting, however, early endoscopic therapy has been shown to reduce mortality and several time-dependent inhospital endoscopic national guidelines exist [8, 43]. The initial assessment by the telephone responder is of great importance in securing efficient prehospital triage, as correct triage will reduce the time from onset of symptoms to initial on scene evaluation by health care professionals. At this point, rapid initial prehospital evaluation of the patient with objective measures of severity will further help clinical decision making and, if necessary, modulate the triage of the patient. Factors that can improve the prehospital care of patients with PPU/PUB include: improvements of the Danish Index, a structured training program for telephone responders in the EMCC, and the development of a fast track handling from the prehospital phase to the inhospital phase in order to improve this transition. Future studies should address these challenges.

## Conclusion

Among patients calling 1-1-2, patients diagnosed with PPU/PUB had a lower proportion of highest level of triage and a higher proportion of lowest level of triage than FHQ patients. The 30-day mortality among patients diagnosed with PPU/PUB was comparable to the 30-day mortality among patients diagnosed with cardiac chest pain and trauma. This study suggests that undertriage is present among patients diagnosed with PPU/PUB.

## Appendix 1

**Table 3:** Stratification of adjusted relative risk on triage and mortality

	Group	Adjusted, RR (95% CI)							
		Sex^a^		Age^b^				CCI Score^c^	
		Male	Female	< 54	54–67	68–79	> 80	0–1	2+
A-triage	PPU/PUB	1 (ref.)	1 (ref.)	1 (ref.)	1 (ref.)	1 (ref.)	1 (ref.)	1 (ref.)	1 (ref.)
	Respiratory Failure	1.12 (0.98–1.29)	1.05 (0.90–1.21)	1.38 (0.97–1.96)	1.08 (0.90–1.31)	1.00 (0.85–1.19)	1.10 (0.91–1.34)	1.10 (0.93–1.30)	1.08 (0.96–1.23)
	Stroke	1.46 (1.28–1.67)	1.34 (1.16–1.55)	1.81 (1.27–2.56)	1.41 (1.18–1.68)	1.29 (1.10–1.52)	1.41 (1.16–1.70)	1.50 (1.27–1.77)	1.34 (1.19–1.52)
	Cardiac Chest Pain	1.52 (1.33–1.73)	1.40 (1.21–1.62)	1.87 (1.32–2.65)	1.45 (1.21–1.73)	1.35 (1.15–1.59)	1.47 (1.21–1.77)	1.58 (1.34–1.86)	1.40 (1.24–1.58)
	Cardiac Arrest	1.49 (1.30–1.70)	1.38 (1.19–1.59)	1.85 (1.31–2.63)	1.41 (1.18–1.70)	1.34 (1.14–1.58)	1.44 (1.19–1.74)	1.53 (1.30–1.81)	1.40 (1.24–1.58)
	Trauma	1.27 (1.10–1.47)	1.30 (1.11–1.52)	1.69 (1.19–2.39)	1.23 (1.01–1.50)	1.10 (0.89–1.35)	1.10 (0.85–1.44)	1.37 (1.15–1.62)	1.17 (1.00–1.36)
E-triage	PPU/PUB	1 (ref.)	1 (ref.)	1 (ref.)	1 (ref.)	1 (ref.)	1 (ref.)	1 (ref.)	1 (ref.)
	Respiratory Failure	0.60 (0.29–1.22)	0.53 (0.23–1.24)	0.31 (0.11–0.90)	0.74 (0.28–1.98)	0.64 (0.22–1.86)	0.75 (0.23–2.44)	0.77 (0.30–2.02)	0.53 (0.28–1.03)
	Stroke	0.39 (0.18–0.82)	0.40 (0.16–0.98)	0.21 (0.06–0.71)	0.24 (0.07–0.82)	0.59 (0.21–1.70)	0.62 (0.18–2.15)	0.48 (1.18–1.28)	0.37 (0.18–0.77)
	Cardiac Chest Pain	0.27 (0.13–0.59)	0.19 (0.06–0.56)	0.10 (0.02–0.46)	0.29 (0.09–0.90)	0.33 (0.10–1.06)	0.31 (0.08–1.20)	0.32 (0.11–0.64)	0.21 (0.10–0.46)
	Cardiac Arrest	0.13 (0.04–0.38)	0.31 (0.10–0.94)	0.12 (0.02–0.75)	0.24 (0.06–1.00)	0.14 (0.03–0.72)	0.17 (0.03–0.99)	0.17 (0.05–0.64)	0.18 (0.07–0.50)
	Trauma	0.16 (0.05–0.53)	0.08 (0.01–0.48)	0.06 (0.01–0.29)	0.17 (0.03–1.02)	–	0.43 (0.04–4.07)	0.13 (0.04–0.46)	0.20 (0.04–0.93)
30-day mortality	PPU/PUB	1 (ref.)	1 (ref.)	1 (ref.)	1 (ref.)	1 (ref.)	1 (ref.)	1 (ref.)	1 (ref.)
	Respiratory Failure	3.31 (1.34–8.14)	1.16 (0.67–2.00)	3.16 (0.26–38.41)	0.88 (0.36–2.20)	1.70 (0.76–3.81)	1.89 (0.90–3.94)	1.40 (0.64–3.10)	1.86 (1.02–3.39)
	Stroke	3.54 (1.46–8.57)	1.53 (0.86–2.71)	14.81 (0.90–242.86)	1.28 (0.52–3.18)	1.84 (0.84–4.04)	2.41 (1.11–5.22)	1.93 (0.88–4.27)	2.20 (1.20–4.04)
	Cardiac Chest Pain	1.23 (0.49–3.10)	0.42 (0.22–0.82)	1.25 (0.07–22.99)	0.10 (0.03–0.36)	0.45 (0.19–1.09)	1.01 (0.46–2.21)	0.35 (0.14–0.88)	0.77 (0.40–1.47)
	Cardiac Arrest	12.39 (5.06–30.09)	5.34 (3.00–9.52)	33.26 (2.01–550.89)	4.65 (2.04–10.61)	7.29 (3.26–16.30)	7.40 (3.49–15.68)	6.75 (3.08–14.80)	6.73 (3.63–12.46)
	Trauma	3.13 (1.14–8.58)	0.65 (0.27–1.59)	2.43 (0.12–50.59)	0.55 (0.18–1.65)	0.93 (0.29–2.96)	2.37 (0.99–5.68)	1.21 (0.49–2.99)	1.31 (0.58–2.92)

*Abbreviations*: *PPU* perforated peptic ulcer, *PUB* peptic ulcer bleeding, *RR* relative risk, *CI* confidence interval *CCI* Charlson Comorbidity Index, *ref.* reference

^a^Adjusted for age, CCI, and time of 1–1-2 call

^b^Adjusted for sex, CCI, and time of 1–1-2 call

^c^Adjusted for age, sex, and time of 1–1-2 call

## Appendix 2

**Table 4:** Imputation on missing data on triage level

Unadjusted A-triage and E-triage				
	Group	Unadjusted, RR (95% CI) Complete Cases	Unadjusted, RR (95% CI) Multiple imputation	Unadjusted, RR (95% CI) Bootstrapping
A-triage (*n* = 7538)	PPU/PUB	1 (ref.)	1 (ref.)	1 (ref.)
	Respiratory Failure	1.10 (0.99–1.22)	1.22 (0.99–1.49)	1.18 (1.06–1.39)
	Stroke	1.41 (1.27–1.56)	1.50 (1.21–1.87)	1.46 (1.29–1.67)
	Trauma	1.32 (1.19–1.46)	1.42 (1.16–1.76)	1.40 (1.25–1.59)
	Cardiac Chest Pain	1.47 (1.33–1.62)	1.58 (1.27–1.96)	1.53 (1.37–1.75)
	Cardiac Arrest	1.46 (1.32–1.61)	1.56 (1.25–1.95)	1.53 (1.33–1.73)
E-triage (*n* = 7538)	PPU/PUB	1 (ref.)	1 (ref.)	1 (ref.)
	Respiratory Failure	0.59 (0.35–1.00)	0.57 (0.31–1.05)	0.65 (0.37–1.48)
	Stroke	0.40 (0.22–0.71)	0.46 (0.24–0.88)	0.54 (0.32–1.11)
	Trauma	0.11 (0.05–0.28)	0.15 (0.06–0.36)	0.16 (0.08–0.40)
	Cardiac Chest pain	0.25 (0.13–0.47)	0.26 (0.12–0.56)	0.33 (0.12–0.85)
	Cardiac Arrest	0.18 (0.08–0.39)	0.18 (0.07–0.45)	0.20 (0.12–0.30)
Adjusted A-triage and E-triage				
	Group	Adjusted, RR (95% CI) Complete Cases	Adjusted^a^, RR (95% CI) Multiple Imputation	Adjusted^a^, RR (95% CI) Bootstrapping
A-triage (*n* = 7538)	PPU/PUB	1 (ref.)	1 (ref.)	1 (ref.)
	Respiratory Failure	1.10 (0.99–1.22)	1.24 (1.01–1.52)	1.20 (1.08–1.42)
	Stroke	1.41 (1.27–1.56)	1.50 (1.21–1.85)	1.45 (1.28–1.67)
	Trauma	1.32 (1.19–1.46)	1.59 (1.29–1.97)	1.57 (1.39–1.79)
	Cardiac Chest Pain	1.47 (1.33–1.62)	1.58 (1.28–1.97)	1.54 (1.37–1.78)
	Cardiac Arrest	1.46 (1.32–1.61)	1.62 (1.29–2.02)	1.58 (1.38–1.81)
E-triage (*n* = 7538)	PPU/PUB	1 (ref.)	1 (ref.)	1 (ref.)
	Respiratory Failure	0.59 (0.35–1.00)	0.52 (0.28–0.95)	0.59 (0.33–1.33)
	Stroke	0.40 (0.22–0.71)	0.48 (0.24–0.93)	0.56 (0.32–1.17)
	Trauma	0.11 (0.05–0.28)	0.10 (0.04–0.25)	0.11 (0.06–0.27)
	Cardiac Chest pain	0.25 (0.13–0.47)	0.25 (0.12–0.54)	0.33 (0.12–0.82)
	Cardiac Arrest	0.18 (0.08–0.39)	0.17 (0.07–0.43)	0.19 (0.12–0.29)

^a^Triage adjusted for age, sex, CCI score, and time of 1–1-2 call

## Appendix 3

**Table 5:** ICD-10 definitions

	Diagnose	ICD-10 codes	Frequency, n (%)
PPU/PUB	Perforated peptic ulcer	DK251x, DK252x, DK255x, DK256x, DK261x, DK262x, DK265x, DK266x, DK271x, DK272x, DK275x, DK276x	216 (2.49)
	Peptic ulcer bleeding	DK250x, DK254x, DK260x, DK264x, DK270x, DK274x	47 (0.54)
Respiratory Failure	Pulmonary embolism	DI26x	208 (2.40)
	Heart failure	DI50.0, DI50.1, DI50.9	407 (4.70)
	Infection	DJ05x, DJ15x, DJ21x, DJ12.9, DJ40.9, DJ42x	616 (7.11)
	Asthma	DJ45x	228 (2.63)
	Pulmonary edema	DJ819	49 (0.57)
	Pneumothorax	DJ93x	84 (0.97)
	Respiratory failure	DJ96.0, DJ96.1, DJ96.9, DR09.2	658 (7.60)
	Dyspnea	DR06.0	339 (3.92)
Stroke	Hemorrhage	DI60x, DI61x, DI62.1, DI62.9	363 (4.19)
	Cerebral infarction	DI63x	720 (8.32)
	Transient cerebral ischemic Attacks and related syndromes	DG45x	605 (6.99)
Cardiac Chest Pain	Angina pectoris	DI20.0, DI20.1, DI20.8, DI20.9	670 (7.74)
	Acute myocardial infarction	DI21x	1121 (12.95)
	Other	DI23x, DI24.9	13 (0.15)
Cardiac Arrest	Cardiac arrest	DI46.0, DI46.1, DI46.9	1083 (12.51)
	Ventricular fibrillation	DI49.0	12 (0.14)
Trauma	Cranio-cerebral trauma	DS021x, DS061, DS062x, DS063x, DS064x, DS065x, DS066x, DS067x, DS068x, DS071x	203 (2.34)
	Thorax injury	DS273x, DS274x, DS277x	2 (0.02)
	Abdominal injury	DS352x, DS367x, DS368x, DS369x	12 (0.14)
	Spine fracture	DS120x, DS127x	22 (0.25)
	Poly-trauma	DS097x, DS383, DS757x, DT025x, DT029x, DT068x	959 (11.08)
	Burns	DT203x, DT213x, DT290x, DT293x, DT312x, DT317x, DT318x	20 (0.23)

*Abbreviations*: *PPU* perforated peptic ulcer, *PUB* peptic ulcer bleeding, *ICD-10* International Classification of Diseases 10th edition, *n* number

## Abbreviations

CCI: Charlson Comorbidity Index
CI: Confidence Interval
CPR: Civil Personal Register
EMCC: Emergency Medical Communication Center
EMS: Emergency Medical Services
FHQ: First Hour Quintet
ICD-10: International Classification of Disease
IQR: Interquartile Ranges
N: Number
OR: Odds Ratio
PPU: Perforated Peptic Ulcer
PUB: Peptic Ulcer Bleeding
Ref.: Reference
RR: Relative Risk
